# Spectral dynamic causal modelling in healthy women reveals brain connectivity changes along the menstrual cycle

**DOI:** 10.1038/s42003-021-02447-w

**Published:** 2021-08-10

**Authors:** Esmeralda Hidalgo-Lopez, Peter Zeidman, TiAnni Harris, Adeel Razi, Belinda Pletzer

**Affiliations:** 1 Department of Psychology and Centre for Cognitive Neuroscience, University of Salzburg, Salzburg, Austria; 2 The Wellcome Centre for Human Neuroimaging, University College London, London, UK; 3 Turner Institute for Brain and Mental Health, Monash University, Clayton, VIC Australia

**Keywords:** Neural circuits, Endocrinology, Neuroscience

## Abstract

Longitudinal menstrual cycle studies allow to investigate the effects of ovarian hormones on brain organization. Here, we use spectral dynamic causal modelling (spDCM) in a triple network model to assess effective connectivity changes along the menstrual cycle within and between the default mode, salience and executive control networks (DMN, SN, and ECN). Sixty healthy young women were scanned three times along their menstrual cycle, during early follicular, pre-ovulatory and mid-luteal phase. Related to estradiol, right before ovulation the left insula recruits the ECN, while the right middle frontal gyrus decreases its connectivity to the precuneus and the DMN decouples into anterior/posterior parts. Related to progesterone during the mid-luteal phase, the insulae (SN) engage to each other, while decreasing their connectivity to parietal ECN, which in turn engages the posterior DMN. When including the most confident connections in a leave-one out cross-validation, we find an above-chance prediction of the left-out subjects’ cycle phase. These findings corroborate the plasticity of the female brain in response to acute hormone fluctuations and may help to further understand the neuroendocrine interactions underlying cognitive changes along the menstrual cycle.

## Abstract (web summary)

Hidalgo-Lopez et al. use dynamic causal modelling to investigate changes in effective connectivity related to menstrual phase and hormonal levels in healthy women. Their results provide further insight into how the brain responds to hormone fluctuations throughout the menstrual cycle.

## Introduction

Physiological fluctuations of ovarian hormones (i.e., estradiol and progesterone) affect the nervous system at multiple levels^1^. Animal research has broadly evidenced the rapid changes exerted by estradiol and progesterone on the neuronal excitatory/inhibitory balance^2^, synaptogenesis^3^, myelination and re-myelination^4^. These effects result in synaptic connectivity changes and therefore neural function^5,6^, and in rodents high levels of both hormones have been shown to improve cognition^7,8^. In ovariectomized rats, both estradiol and progesterone treatment enhances novel object recognition, mechanistically linked to increased long-term potentiation and N-methyl-D-aspartic acid receptors at hippocampal synapses for estradiol^7^, and to an increased density of the basal dendrites of hippocampal neurons for progesterone^8^. More importantly, as evidenced in hormonal replacement studies in rodents^9^ and non-human primates^10–12^ at least some of the reported neuroactive effects are dependent on a cyclic variation of hormone levels, as opposed to a continuous schedule of treatment. Although in humans such multilevel relationships between molecular mechanisms and neural functions are more difficult to determine, new results hint that neural reorganization attributed to the hormones also appears abolished when the endogenous fluctuation of ovarian hormones is disrupted^13–15^. This emphasizes the importance of studying menstrual cycle-related neural changes in healthy subjects, which still remains underinvestigated. Although sparse, acute structural changes related to hormonal fluctuations have already been reported for both grey^16,17^ and white matter^18^. Accordingly, connectivity patterns vary in women depending on the hormonal status, as shown by resting-state functional MRI (fMRI)^19–21^.

Amongst the most common approaches to resting-state functional MRI is the assessment of intrinsic connectivity networks (ICNs). These sets of brain networks with temporally correlated activity at rest relate to task-based BOLD activation patterns^22,23^ and are consistent across healthy subjects^24,25^. Three large-scale systems derived by independent component analyses (ICA) arise as ‘core’ neurocognitive networks, essential for cognitive functions^26^. First, the default mode network (DMN), which includes the precuneus/posterior cingulate cortex (PCC), medial prefrontal cortex (mPFC) and bilateral angular gyrus (AG), is characterized by increased activity during the resting state and decreased activity during goal-directed tasks^27,28^. Second, the salience network (SN), which comprises bilateral anterior insula (AI) and dorsal anterior cingulate cortex (ACC), is specialized in identifying and mapping relevant inputs, such as emotional stimuli^29,30^. Third, the executive control network (ECN), is composed of bilateral middle frontal gyri (MFG) and bilateral supramarginal gyri (SMG)^30^. This fronto-parietal system is usually lateralized^23,24^ and responsible for higher cognitive control functions, such as working memory or directed attention, once the relevant stimuli are detected^25,30^.

Although some studies have reported no menstrual cycle-related effects on resting-state functional connectivity^31,32^, all of these ICNs are susceptible to modulation by ovarian hormones. Specifically, within the DMN, intrinsic resting-state connectivity has been shown to increase before ovulation^16,33^ and decrease during the luteal phase to both left and right AG^21,34^. More recently, a single-subject study showed that the peak in estradiol right before ovulation had a key role in temporarily reorganizing the functional coupling of ICNs, especially within-network connectivity of DMN and dorsal attention network^14^. Further analyses in this dataset showed that during this time window several brain nodes increased their flexibility, defined as a measure of how often each node changes its affiliation among different networks or functional modules. Specifically, limbic and subcortical nodes were more flexible around ovulation and the mid-luteal phase, while the posterior DMN (PCC) was most flexible around ovulation, followed by an increased stability after the estradiol’s peak^13^. Within the SN, higher activity and connectivity have been consistently reported during the luteal cycle phase and related to higher progesterone levels, both at rest and during tasks^35,36^. Finally, for the ECN, increased task-based activation with higher levels of estradiol and progesterone have been shown for frontal areas. Specifically, increased right dorsolateral PFC activation has been reported during the luteal phase, over a range of tasks including spatial and verbal working memory and verbal fluency^37–40^. Relatedly, during resting state, increased eigenvector centrality in bilateral dorsolateral PFC has been found in the presence of higher progesterone levels^41^.

Regarding inter-network connectivity, previous findings mainly report changes in the DMN-SN connectivity, pointing out an increase during the luteal phase (however, not without inconsistencies, see ref. ^33^). Specifically, ACC and amygdala (nodes of the SN) increased their connectivity with the precuneus (posterior DMN) during the luteal phase compared to early follicular^42^. Likewise, when treated with progesterone, increased connectivity between several nodes of the SN-DMN has been reported^35^. This enhanced inter-connectivity between SN and DMN, alongside the increased intrinsic connectivity in the SN and decreased intrinsic connectivity in the DMN, sets a very unique scenario for the luteal phase. Considering that both networks involved (specially ACC and mPFC) are also implicated in self-referential^43^ and affective experience^28,44^, this distinctive connectivity pattern could elicit the misattribution of salient stimuli and dysfunctional appraisal, leading to anxiety and depressive symptoms^26^. Consequently, the coupling dynamics between and within the SN and DMN have been proposed to underlie the window of vulnerability for cycle-related affective disorders during the luteal phase^36,45^. More importantly, the precise role of the ECN, responsible for the regulation of the SN and the DMN, has not yet been defined. Weis et al.^46^ observed decreased connectivity during the pre-ovulatory phase between the DMN and the left MFG (part of the ECN), while Petersen et al.^34^ observed decreased connectivity during the luteal phase between the ECN and the ACC (part of the SN), both compared to early follicular. These changes could imply a difference in functional integration of cognitive and affective processes, but up to now a cohesive model for understanding the global mechanisms in which the healthy female brain adapts to hormonal changes remains elusive.

Despite being a useful resource to address brain organization, the assessment of resting-state functional connectivity^23,25,28,47^ does not allow to infer the directionality of coupling between these multiple distributed systems. Effective connectivity methods, on the other hand, constitute the best approach to investigate the complex within and between ICN relationships and the causal influence that one node exerts over another. Specifically, model inversion with spectral dynamic causal modelling (spDCM) estimates hidden neural states from the observed Blood Oxygen Level Dependent (BOLD) signal—specifically, the cross-spectral density of BOLD signals from different brain regions. It assumes that spontaneous fluctuations in the signal during resting state reflect the endogenous neural activity^48^. By parameterising the hidden coupling among the neuronal populations, one can generate (complex) cross spectra among observed responses^49–51^. In addition, spDCM is especially efficient to invert large DCMs of resting-state fMRI^51^, which makes it feasible to assess large-scale between and within networks’ organization.

To the best of our knowledge, no study has yet investigated the directed connectivity across the different menstrual cycle phases and related to ovarian hormonal levels. In this work, we aim to delineate the menstrual cycle-related changes in effective connectivity within and between DMN, SN and ECN, and identify the specific direction of previously reported effects^16,34–36,42,52^. For example, it is yet not defined whether the enhanced inter-connectivity of the SN and DMN during the luteal cycle phase^42^ originates from the SN or the DMN. Likewise, it is unclear whether the downregulation of the SN during the luteal phase is related to an increase in the directed connectivity from the ECN as proposed by the triple network model^26^. In addition, it also remains unexplored how the increased activation of the right MFG relates to increased afferent connectivity, given that the BOLD-response rather reflects the input to a neuronal population^53^. As several psychiatric and neurological disorders share an aberrant intrinsic organization of the aforementioned three core networks^26^, we consider it of the utmost importance to characterize their non-pathological directed organization in healthy women. In order to do so, we applied state-of-the-art effective connectivity analyses to a previously published longitudinal resting state-fMRI data set acquired during early follicular, pre-ovulatory and mid-luteal cycle phase in a large sample of healthy young women^21^. Finally, we aimed to identify, whether changes in specific directed connections were able to predict which cycle phase a woman was in.

## Results

### Demographic and hormonal data

For the final sample and as expected, both estradiol and progesterone levels significantly changed within subject across the menstrual cycle (F2, 114 = 29.02, *p* < 0.001; F2, 114 = 33.49, *p* < 0.001, for estradiol and progesterone respectively) (Table 1). For estradiol, levels were significantly higher during the pre-ovulatory phase compared to early follicular (SE =  0.09, *t* = 7.61, *p* < 0.001) and mid-luteal phase (SE = 0.10, *t* = −3.01, *p* < 0.01), as well as during the mid-luteal phase compared to early follicular (SE = 0.10, *t* = 3.49, *p* < 0.01). Progesterone levels were significantly lower during early follicular compared to the pre-ovulatory and the mid-luteal phase (SE = 0.06, *t* = 3.19, *p* < 0.01; SE = 0.15, *t* = 8.12, *p* < 0.001, respectively), and higher during the mid-luteal compared to the pre-ovulatory phase (SE = 0.13, *t* = 7.82, *p* < 0.001).

**Table 1:** Demographic data and hormone levels during each cycle phase.

Sample (*n* = 58)	Age (years)	APM (IQ)	Cycle length (days)	Cycle day of assesment (days)	Estradiol (pg/ml)	Progesterone (pg/ml)
EF	25.36 ± 0.56	110.31 ± 1.21	28.22 ± 0.31	3.72 ± 0.20	0.81 ± 0.05	65.22 ± 5.3
P-O				12.02 ± 0.32	1.11 ± 0.07^***^	86.98 ± 8.02^**^
M-L				21.32 ± 0.47	0.97 ± 0.06^**^	199.06 ± 18.28^***^

Values are presented as mean ± standard error of the mean (M ± SEM) for the final sample of *n* = 58.

*EF* early follicular, *P-O* pre-ovulatory, *M-L* mid-luteal.

For hormone levels, ** corresponds to *p* < 0.01, and *** corresponds to *p* < 0.001, compared to early follicular.

### Spectral dynamic causal modelling and parametric empirical bayes

**Fig. 1:**
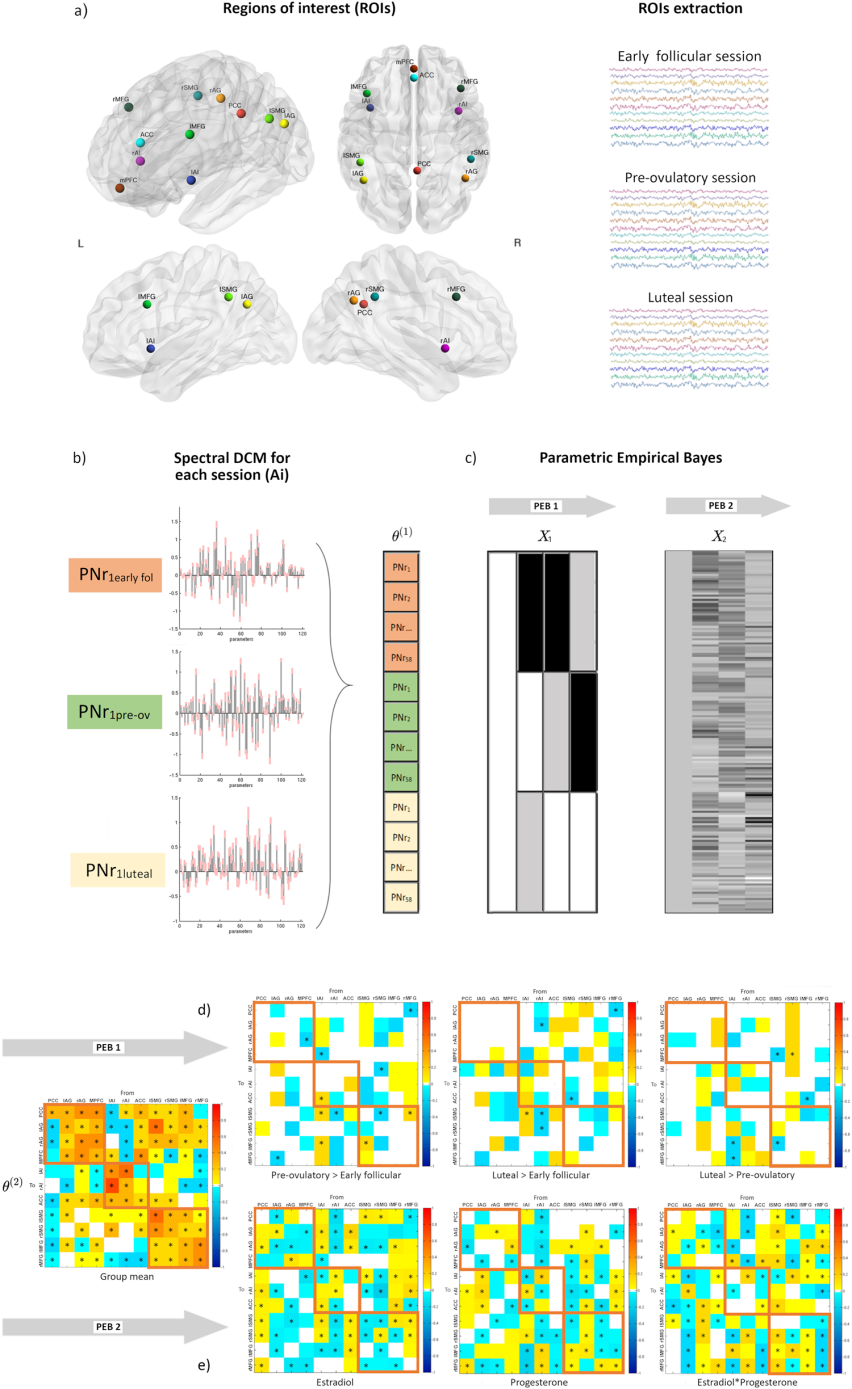
Procedures for dynamic effective connectivity analysis. The regions of interest from each ICN used in the current study is shown in (**a**). The default mode brain regions included the precuneus/posterior cingulate cortex (PCC), medial prefrontal cortex (mPFC) and bilateral angular gyrus (AG); the salience network comprised bilateral anterior insula (AI) and anterior cingulate cortex (ACC), and the executive control network was composed of bilateral middle frontal gyri (MFG) and bilateral supramarginal gyri (SMG). Each participant had three sessions locked to their menstrual cycle: during early follicular, pre-ovulatory and mid-luteal phase. **b** A spectral DCM (spDCM) of 121 parameters was estimated for each session of every participant in a group DCM (θ_1_). **c** For the group-level analysis, Parametric Empirical Bayesian analysis (PEB) was used to investigate the cycle phase and hormonal levels group effects. This is a general linear model of the connectivity parameters. Shown are the design matrix X_1_ for cycle phase and X_2_ for hormonal levels, where lighter colours indicate higher values. **d** Estimated parameters for PEB 1 (cycle phases). **e** Estimated parameters for PEB 2 (hormonal levels). For (**d**) and (**e**) connections surpassing a posterior probability of 95% are indicated with an asterisk. The columns are the outgoing connections, the rows are the incoming connections, ordered as: PCC, lAG, rAG, mPFC, lAI, rAI, ACC, lSMG, rSMG, lMFG, and rMFG. Hot colours indicate positive parameter estimates and cold colours negative.

Results are displayed in Fig. 1d, e, 2 (within-network) and 3 (between-network) and reported in Supplementary Data file 1. Only parameters showing a ‘positive evidence’^54^ (more than 75% posterior probability of having diverged from its prior expectation of zero) are reported. Those connections surviving a 95% threshold are further indicated in the figures and results section alongside each parameter’s estimated mean (Ep) and posterior probability (Pp). Given that all parameters contributed to the model, figures including all parameters regardless of threshold can be found in the supplementary material (SI, Fig. S2 cycle phase results, and Fig. S3 hormonal results). Most of the changes in cycle phase were backed up by hormonal correlations (compare Supplementary Data file 1), but only those surviving a 95% threshold are reported in the text.

### Within-network effective connectivity

**Fig. 2:**
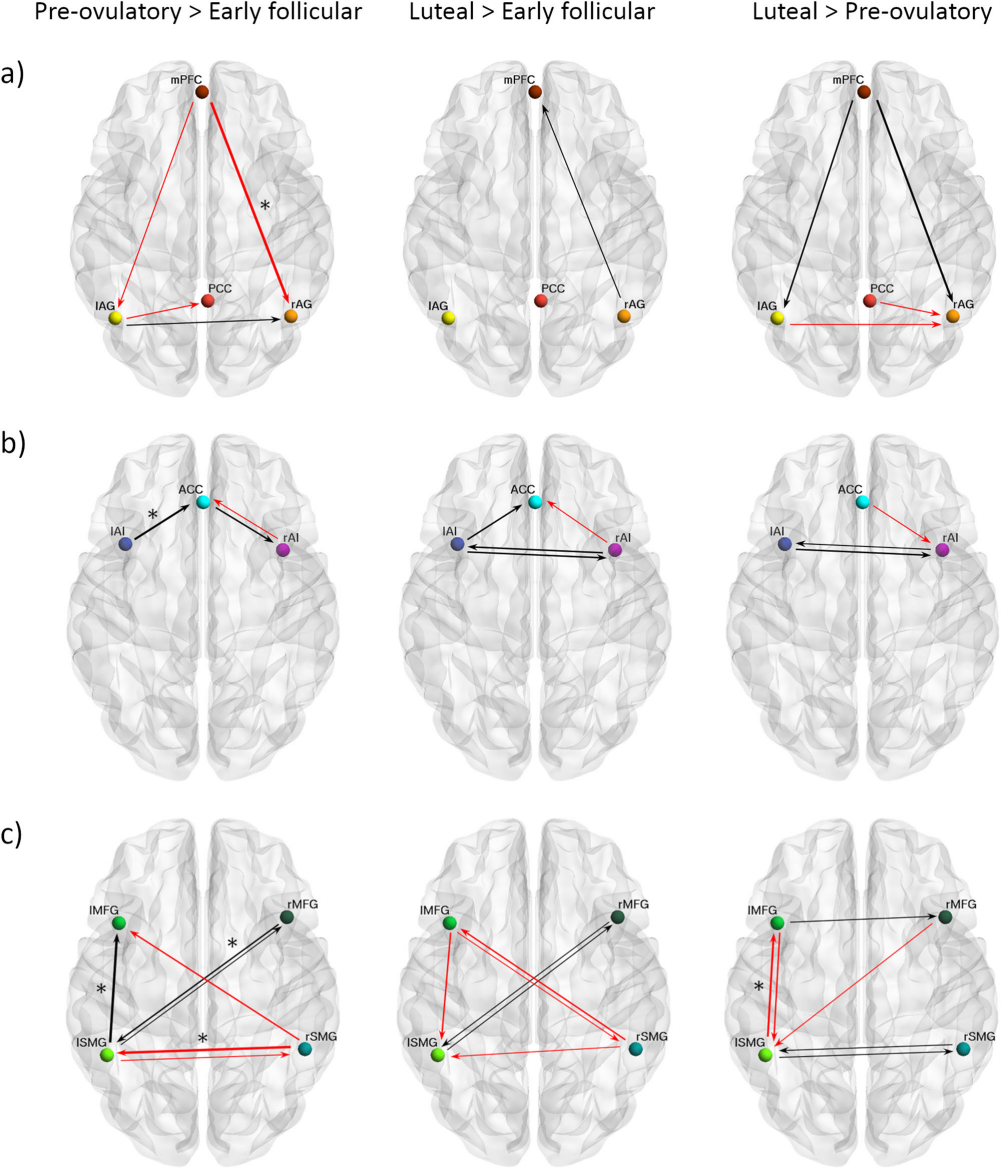
Cycle phase differences in within-network effective connectivity. **a** DMN; **b** SN; **c** ECN. Only connections with a posterior probability >0.75 are displayed. Connections surpassing a posterior probability of 95% are indicated with an asterisk. The results reflect connection strengths as a difference between the indicated cycle phases. The differential connection strengths are depicted by the width of the arrow. Black arrows reflect positive values and red arrows reflect negative values for those connections which showed differences in the former than in the latter indicated cycle phase.

DMN: In general, effective connectivity within anterior and posterior nodes in the DMN was highest during early follicular and lowest during the pre-ovulatory phase (Supplementary Data file 1, Fig. 2a, −0.14 < Ep < 0.11, Pp > 75%). Specifically, effective connectivity from mPFC to right AG (Ep = −0.14, Pp = 96%) decreased from early follicular to the pre-ovulatory phase, negatively related to estradiol levels (Ep = −0.06, Pp = 95%). This negative impact of higher estradiol levels was reversed by progesterone positive impact (Ep = 0.12, Pp = 100%), resulting in an interactive effect of both hormones (Ep = −0.06, Pp = 96%) (Supplementary Data file 1, Fig. 2a).

SN: Within the SN bidirectional connectivity between left and right AI was strongest during the mid-luteal phase (Supplementary Data file 1, Fig. 2b, 0.07 < Ep < 0.09, Pp > 75%). While effective connectivity of the right AI to the ACC decreased during the pre-ovulatory and mid-luteal phase compared to early follicular, effective connectivity from the left AI to the ACC followed the opposite pattern (Ep = 0.12, Pp = 95%). Effective connectivity from the left AI to ACC was further positively related to the increase in estradiol levels (Ep = 0.08, Pp = 100%), and negatively related to progesterone levels (Ep = −0.06, Pp = 98%)(Supplementary Data file 1, Fig. 2b).

ECN: Within the ECN, in general, connectivity between homotopic areas was weakest during the pre-ovulatory phase (Supplementary Data file 1, Fig. 2c, −0.15 < Ep < 0.15, Pp > 75%). Specifically, effective connectivity from right to left SMG decreased from early follicular to the pre-ovulatory phase (Ep = −0.15, Pp = 100%), and was negatively related to estradiol levels (Ep = −0.09, Pp = 100%) (Supplementary Data file 1, Fig. 2c). Conversely, from right MFG to left SMG effective connectivity increased from early follicular to pre-ovulatory phase (Ep = 0.10, Pp = 98%), positively related to estradiol levels (Ep = 0.09, Pp = 100%). The positive impact of estradiol was reduced in the presence of high progesterone levels, as indicated by a negative interaction effect (Ep = −0.04, Pp = 99%) (Supplementary Data file 1). In general, connectivity between the left-lateralized nodes was the strongest right before ovulation (Fig. 2c). Specifically, effective connectivity from the left SMG to the left MFG increased from early follicular to the pre-ovulatory phase (Ep = 0.15, Pp = 100%), and decreased again during the mid-luteal phase (Ep =  −0.12, Pp = 99%). For this connection, higher progesterone (Ep = 0.05, Pp = 99%) and the combined effect of estradiol and progesterone (Ep = 0.05, Pp = 100%) had a positive impact.

### Between-network effective connectivity

**Fig. 3:**
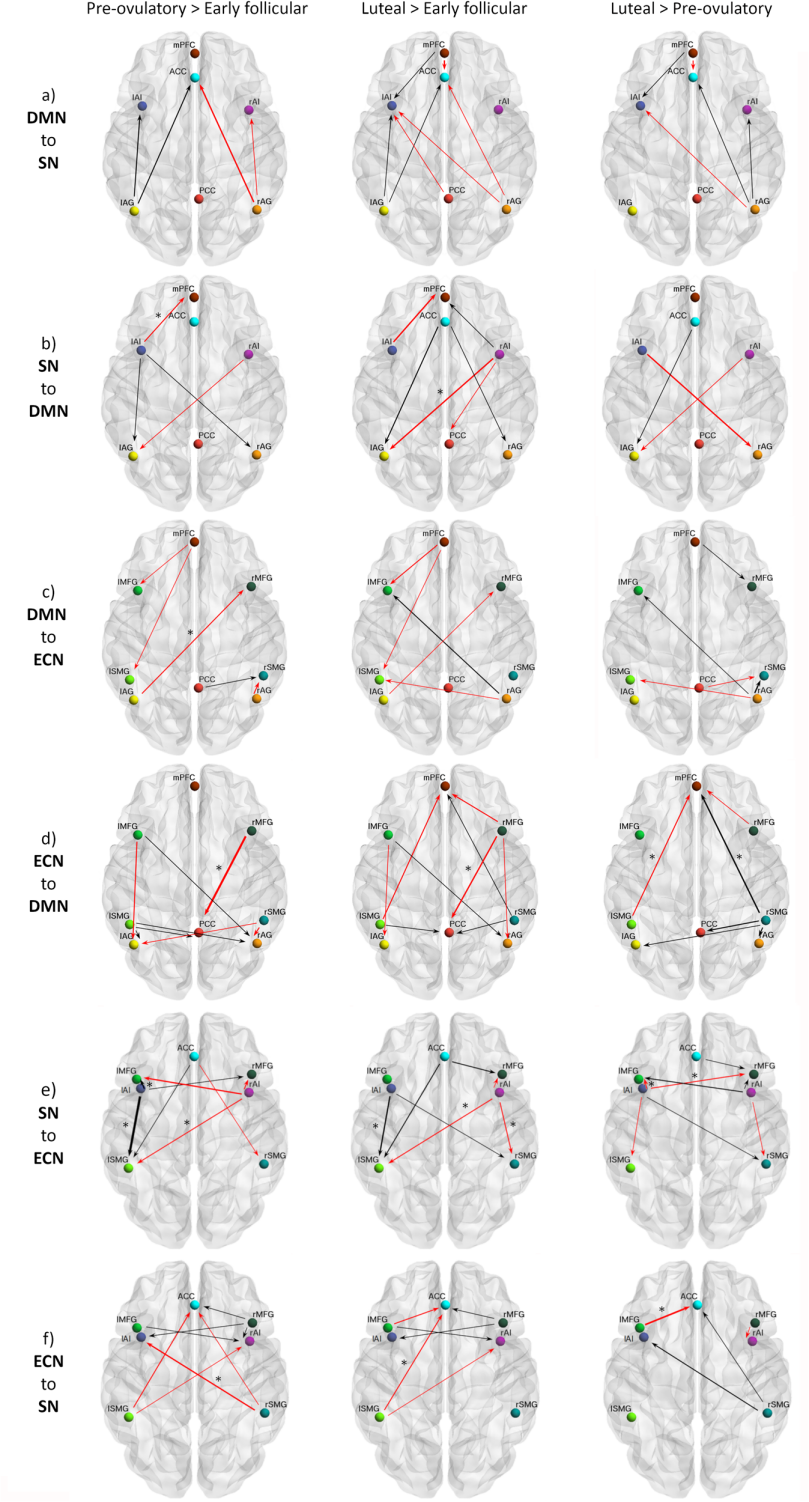
Cycle phase differences in between-network effective connectivity. **a** From the DMN to the SN; **b** From the SN to the DMN; **c** From the DMN to the ECN; **d** From the ECN to the DMN; **e** From the SN to the ECN; **f** From the ECN to the SN. Only connections with a posterior probability >0.75 are displayed. Connections surpassing a posterior probability of 95% are indicated with an asterisk. The results reflect connection strengths as a difference between the indicated cycle phases. The differential connection strengths are depicted by the width of the arrow. Black arrows reflect positive values and red arrows reflect negative values for those connections which showed differences in the former than in the latter indicated cycle phase.

DMN—SN: Effective connectivity between the DMN and SN across the menstrual cycle was characterized by a lateralized pattern. From left AG to ipsilateral AI and ACC was stronger during the high-hormone phases, while from right AG to ipsilateral AI and ACC, connectivity was weakest during the pre-ovulatory phase. Furthermore, during the mid-luteal phase, connectivity from the mPFC to left AI was the strongest, whereas to the ACC, the lowest (Supplementary Data file 1, Fig. 3a, −0.12 < Ep < 0.09, Pp > 75%). In general, effective connectivity from the SN to bilateral AG was strongest during the high-hormone phases, except for those connections originating in the right hemisphere. Specifically, from right AI to left AG it decreased from early follicular to the mid-luteal phase (Ep = −0.11, Pp = 95%) and was negatively correlated to estradiol (Ep = −0.01, Pp = 100%), progesterone (Ep = −0.05, Pp = 98%) and their combinatory effect (Ep = −0.17, Pp = 100%) (Supplementary Data file 1, Fig. 3b). The effective connectivity from the SN to the anterior DMN followed an opposite lateralization, being the strongest during the early follicular phase from the left AI to mPFC (Ep = −0.11, Pp = 95%), and negatively related to estradiol (Ep = −0.05, Pp = 97%). The negative impact of higher estradiol levels was partially reversed in the presence of high progesterone levels as indicated by a positive interaction effect (Ep = 0.10, Pp = 100%) (Supplementary Data file 1).

DMN—ECN: In general, effective connectivity from the DMN to the ECN was left-lateralized during early follicular, whereas right-lateralized during the mid-luteal phase, and the lowest right before ovulation (Supplementary Data file 1, Fig. 3c, −0.10 < Ep < 0.11, Pp > 75%). From the left AG to right MFG, effective connectivity decreased from early follicular to the pre-ovulatory phase (Ep = −0.11, Pp = 97%) and related negatively to progesterone levels (Ep = −0.10, Pp = 100%). This effect was further enhanced in the presence of high estradiol levels (Ep = −0.12, Pp = 100%) (Supplementary Data file 1, Fig. 3c). In turn, effective connectivity from the posterior nodes of the ECN to the posterior DMN increased from early follicular to the mid-luteal phase (Supplementary Data file 1, Fig. 3d, 0.06 < Ep < 0.10, Pp > 75%). During this phase, effective connectivity to the mPFC, was the highest from the right SMG (Ep = 0.11, Pp = 95%), whereas the lowest from the left SMG (Ep = −0.10, Pp = 96%). Accordingly, these effects were positively (Ep = 0.14, Pp = 100%) and negatively related to progesterone levels (Ep = −0.05, Pp = 99%), but reversed in the presence of high estradiol (right SMG: Ep = −0.11, Pp = 100%, left SMG: Ep = 0.10, Pp = 100%) (Supplementary Data file 1). Conversely, during early follicular, connectivity was the strongest from frontal ECN to the DMN (Fig. 3.d). Specifically, from the right MFG to PCC, effective connectivity decreased during the pre-ovulatory (Ep = −0.17, Pp=99%) and the mid-luteal phase (Ep = −0.13, Pp = 97%), and related negatively to estradiol levels (Ep = −0.08, Pp = 100%) (Supplementary Data file 1).

SN—ECN: Effective connectivity changes from the SN to the ECN were also strongly lateralized. In general, during the early follicular phase effective connectivity was the strongest from the right AI while lowest from the left AI, whereas during the pre-ovulatory phase the opposite pattern was observed (Supplementary Data file 1, Fig. 3e, −0.14 < Ep < 0.20, Pp > 75%).

On one hand, from the left AI to the left SMG effective connectivity was the lowest during early follicular compared to the pre-ovulatory (Ep = 0.20, Pp = 100%), and mid-luteal phases (Ep = 0.13, Pp = 99%), and positively related to estradiol levels (Ep = 0.11, Pp = 100%) (Supplementary Data file 1). Likewise, effective connectivity from the left AI to the left MFG also increased from the early follicular to the pre-ovulatory phase (Ep = 0.12, Pp = 96%). During the mid-luteal phase, effective connectivity from the left AI to frontal ECN decreased again (left MFG: Ep = −0.15, Pp = 99%; right MFG: Ep = −0.10, Pp = 96%). Accordingly, both connections were negatively correlated to progesterone levels (left MFG: Ep = −0.18, Pp = 100%; right MFG: Ep = −0.20, Pp = 100%). Likewise, higher estradiol levels had a negative impact on the connectivity from the left AI to the left MFG (Ep = −0.10, Pp = 100%), while the combinatory effect with progesterone was positive (Ep = 0.10, Pp = 100%). From the left AI to the right MFG, connectivity was further negatively affected by the combinatory effect of estradiol and progesterone (Ep = −0.12, Pp = 100%)(Supplementary Data file 1).

On the other hand, from the right AI, effective connectivity to posterior ECN decreased right before ovulation (Ep = −0.09, Pp = 96% to the left SMG), and during the mid-luteal phase (Ep = −0.11, Pp = 97% to the left and Ep = −0.11, Pp=95% to the right SMG) and was related to higher estradiol (left SMG: Ep = −0.13, Pp = 100%; right SMG: Ep = −0.07, Pp = 99%), lower progesterone (left SMG: Ep = −0.06, Pp = 100%; right SMG: Ep = −0.11, Pp = 100%) and their combinatory effect (right SMG: Ep = 0.09, Pp = 100%) (Supplementary Data file 1, Fig. 3e).

Effective connectivity from the frontal ECN to the SN was in general increased during the high-hormone phases (0.04 < Ep < 0.08, Pp > 75%), except from the left MFG to the ACC, which decreased from the pre-ovulatory to the mid-luteal phase (Ep = −0.15, Pp = 97%), and was negatively related to progesterone levels (Ep = −0.15, Pp = 100%), and positively to estradiol (Ep = 0.15, Pp = 100%) (Supplementary Data file 1, Fig. 3f).

Effective connectivity from bilateral SMG to ACC and each contralateral AI decreased from early follicular to the pre-ovulatory phase. Specifically, from the right SMG to the left AI, effective connectivity decreased right before ovulation (Ep = −0.11, Pp = 96%), and related negatively to estradiol levels (Ep = −0.10, Pp = 100%). The negative impact of higher estradiol levels was reversed by progesterone’s positive impact (Ep = 0.06, Pp = 100%), as indicated by a significant interaction effect (Ep = 0.08, Pp = 100%) (Supplementary Data file 1). During the mid-luteal phase, only those connections originating in the right hemisphere increased again in connectivity strength, while connections originating from the left hemisphere stayed decreased (Ep = −0.10, Pp = 95% from left SMG to ACC) and were negatively related to progesterone levels (Ep = −0.10, Pp = 100%). The latter effect was partially reversed in the presence of high estradiol, as indicated by a significant interaction effect (Ep = 0.12, Pp = 100%) (Supplementary Data file 1, Fig. 3f).

### Leave-one-out cross-validation (LOOCV)

**Fig. 4:**
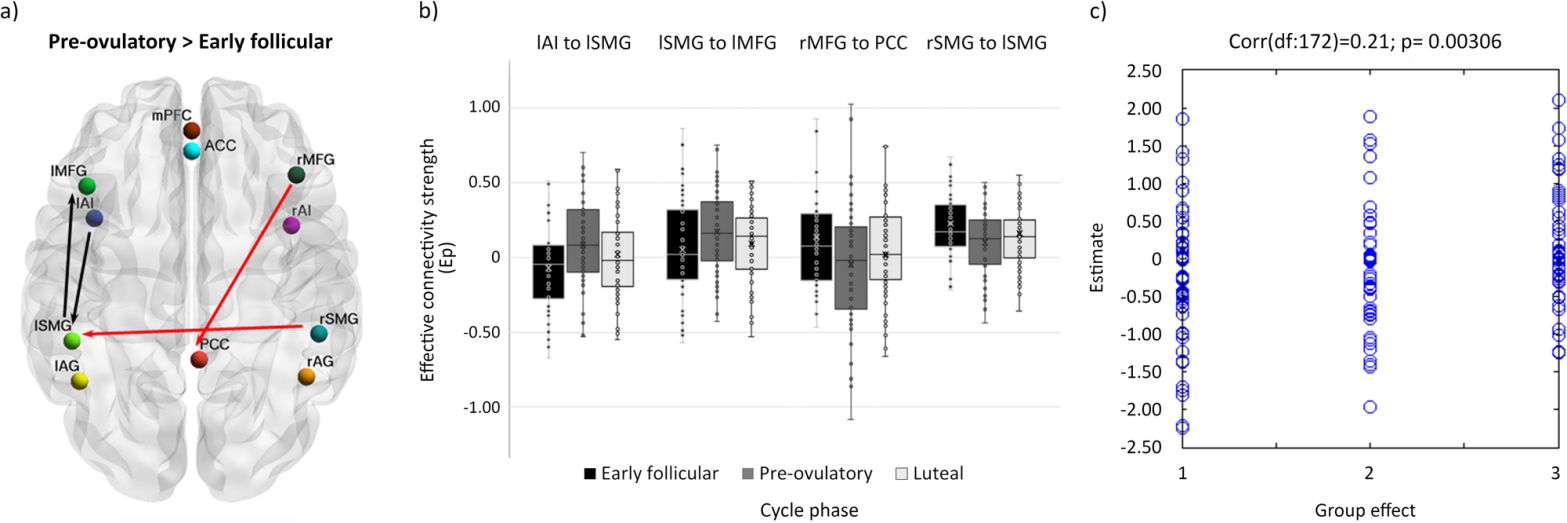
Cycle phase differences in effective connectivity within and between intrinsic connectivity networks DMN, SN and ECN with a posterior probability >0.99. **a** Differential connectivity strength between the indicated cycle phases is depicted by the width of the arrow. Black arrows reflect positive values and red arrows reflect negative values for those connections which showed differences in the former than in the latter indicated cycle phase. **b** Box plot showing parameter estimates per cycle phase for each connection, *n* = 174. **c** Out-of-samples correlation scatter plot from the LOOCV analysis displaying the correlation between the actual cycle phase in the left-out-subject’s design matrix (early follicular, pre-ovulatory or mid-luteal) and the predicted cycle phase based on the left-out-subject’s connectivity (*r*_df:172_ = 0.21, *p* = 0.003). Source data can be found in Supplementary Data file 2 and 3, respectively.

We assessed whether the individual cycle phase could be predicted based on the modulation of effective connectivity between those areas that survived a threshold of posterior probability >0.99 (‘very strong evidence’^54^) in the previous analyses. Those directed connections were from left AI and right SMG to left SMG, from left SMG to left MFG, and from right MFG to PCC. The Pearson’s correlation coefficient between the actual cycle phase in the left-out-subject’s design matrix (early follicular, pre-ovulatory or mid-luteal) and the predicted cycle phase based on the left-out-subject’s connectivity was rdf:172 = 0.21, *p* = 0.003 (Fig. 4). Thus, the difference across the menstrual cycle in effective connectivity between these areas was sufficiently large to predict the left-out subject’s cycle phase above chance, although there is still a lot of variability to be explained.

## Discussion

**Fig. 5:**
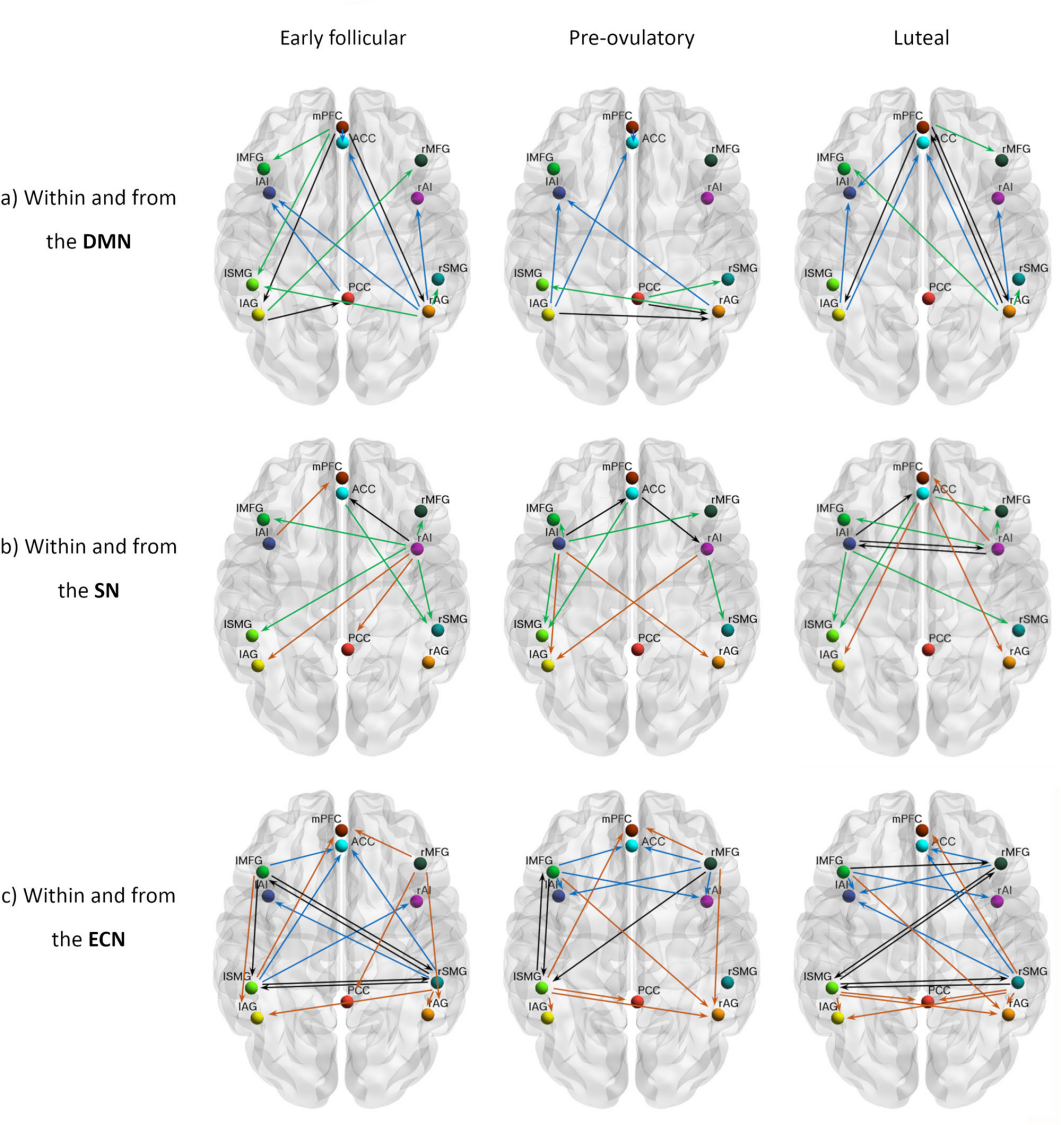
Summary of cycle-related differences in within and between-network effective connectivity. Each row depicts the connections observed to be enhanced in each cycle phase compared to the others within and from (**a**) DMN; (**b**) SN; and (**c**) ECN. Only connections with a posterior probability >0.75 are displayed. Within-network connections are depicted in black and the efferent connectivity to the DMN in brown, to the SN in blue, and to the ECN in green.

Research on the brain organization of naturally cycling women is of the utmost importance for understanding the neurobiological underpinnings of cognitive and emotional effects of ovarian hormones. However, to the best of our knowledge no prior studies have longitudinally assessed the resting-state effective connectivity related to the endogenous hormone fluctuations. Therefore, we used spectral DCM to characterize the temporal dynamics of brain connectivity in a triple network model across the natural menstrual cycle. Overall, some distinct patterns arose in each cycle phase and distinctively for each network, which will be discussed in detail in the following paragraphs and are depicted in Fig. 5. In summary, during the early follicular phase, characterized by low levels of estradiol and progesterone, we observed increased right lateralization of efferent connectivity from the SN and DMN, increased integration within the DMN and between DMN and ECN, and a higher recruitment of the SN by the parietal ECN. Then, right before ovulation, the lateralization shifted, as the left insula increased its efferent connectivity in response to heightened estradiol levels. It recruited the fronto-parietal network, which caused the right MFG to decouple from PCC. In exchange, the PCC engaged to bilateral AG, decoupling the DMN into anterior/posterior parts. Finally, during the mid-luteal phase, the SN increased the connectivity within its own network, recruiting the right hemisphere again, and affecting differentially the other networks depending on the lateralization. Now the right insula recruited the frontal areas of the other networks. In turn, frontal nodes of ECN maintained the enhanced connectivity to the SN, while its posterior nodes increased their connectivity to the posterior DMN. In general, after ovulation, lateralization decreased as the homotopic regions of the ECN and SN were more connected to each other.

First, the present findings demonstrate an anterior-posterior modulation not only within the DMN but also in the connectivity between the DMN and the other networks. The global integration of the DMN was highest during menses, with enhanced connections from and within the entire DMN. During this phase, frontal ECN, particularly the left MFG, was most coupled with the DMN, in line with Weis et al.^46^. Right before ovulation, the bilateral AG disconnected from frontal areas and engaged to the PCC and to each other, increasing the connectivity within the posterior DMN. After ovulation, the AG switched again their connectivity to anterior areas disengaging from the PCC, and connections from the parietal ECN increased, especially to the posterior part of the DMN. The increased connectivity between bilateral AG and anteromedial areas during the mid-luteal phase accompanied a decreased integrity of the posterior DMN, as the PCC decoupled from the DMN and was recruited by the parietal ECN. Remarkably, these findings suggest that the decomposition of DMN into anterior and posterior components, often described in ICA-analyses^24,55^ at least in women, respond to hormonal factors. Relatedly, the PCC has been already identified not only as a highly interconnected node that may functionally switch between different brain networks^56^, but also as the most flexible node around the ovulatory window^13,14^. In Mueller et al. study^13^, around ovulation, the PCC increased its functional connectivity to other nodes, including prefrontal areas related to the DMN, not included in our model. The present results corroborate estradiol’s suggested role as a facilitator for this function^13,14^, and extend previous work by adding the direction in which its levels affect the PCC connectivity, being mostly the efferent connections to the other networks the ones positively related to estradiol levels (Fig. 1e).

The hypothesized increased connectivity between the DMN and SN^36^ was only corroborated partially, given the anterior/posterior and lateralized pattern, and probably reflects an unbalanced mechanism rather than its organization in the non-pathological brain. Women with a history of affective side effects of the pill showed enhanced ACC-PCC connectivity both during treatment and the mid-luteal phase compared to menses (not pill-active)^42^. Furthermore, the over recruitment of the ACC into the DMN and enhanced connectivity with the mPFC has been characterized as unique for major depression symptoms and could reflect an inability to attend salient relevant external stimuli^26^. Conversely, in our model we observed a reduced connectivity from mPFC to ACC during the mid-luteal phase, related to higher progesterone levels, while increased mPFC-PCC coupling to parietal areas, involved in external information processing (Fig. 1e, Fig. 5). Thus, we suggest that these coupling-decoupling effects respond to a compensatory mechanism in healthy women, and its absence could underlie the vulnerability to affective symptoms described during the luteal phase^36,57^.

Second, these results suggest that the insula’s role as a switcher between networks may also respond to hormonal factors. Increased reactivity of the SN, crucial for detecting emotional saliency, along with the deregulated coupling to frontoparietal systems has been proposed to underlie the vulnerability to develop affective disorders, in general^26^, and particularly during the luteal phase^36,45,57^. As expected, we found an increased within SN connectivity during the mid-luteal phase related to higher hormone levels, reflected in the enhanced inter-hemispheric connectivity between the insulae (Figs. 1e, 5). Increased resting-state functional connectivity between the insular cortices has already been related to higher estradiol levels in girls with precocious puberty^58^ and higher flexibility of the limbic system concurrent to increased estradiol and progesterone levels, effect absent when the menstrual cycle was disrupted^13^. As a core node strongly and reciprocally connected to widespread cortical and subcortical areas^59^, the insular cortex orchestrates interoception, cognition and emotion, contributing to emotional awareness, learning and memory processes^59–61^. This unique position allows the AI to engage to the ECN as it disengages from the DMN, acting as switcher between networks^62–64^.

The present results not only corroborate these previous findings in causal networks’ dynamics^62–64^, but also suggest a further aspect of the insula’s function related to its role in responding to changes in the endogenous hormonal milieu. Indeed, previous studies have already related the insula’s morphology and resting state connectivity to women’s hormonal status in a lateralized pattern^65,66^. In our sample, the left insula had higher connectivity to the anterior DMN during early follicular, while the right insula was more strongly connected to the posterior DMN and ECN. Then, during the pre-ovulatory phase related to the increased estradiol levels, this pattern reversed and the left insula increased its connectivity to posterior DMN, ACC and ECN, as the right insula decreased it (Supplementary Data file 1). Although the bilateral AI are involved in the response to all emotional stimuli, a left-hemisphere dominance for positive stimuli^67^ and empathy^68^ has been suggested, relating the lateralized pattern of insula coupling and decoupling to the ECN to menstrual cycle-dependent changes in emotion processing^69^. During the mid-luteal phase both insulae decoupled from the PCC as they increased the connectivity to each other. In addition, the left insula maintained the connectivity with parietal ECN while the right insula replaced its counterpart recruiting again the frontal ECN. Accordingly, these findings support the idea that the enhanced salience detection during the mid-luteal phase could be buffered by coupling with frontal ECN. In fact, the increased connectivity between ACC and right MFG during the mid-luteal phase may reflect an enhanced top-downregulation in healthy women.

Third, the ECN showed both a lateralized and anterior-posterior hormonal modulation, in line with previous neuroendocrine research. Converging evidence from animal and human research relates estradiol to improved prefrontal-dependent functions, especially in verbal tasks (for a review, see ref. ^5^). In women, frontal areas such as the IFG and MFG have shown enhanced activity during the pre-ovulatory phase and related to increased estradiol levels for implicit memory^70^ and verbal encoding^71^. We recently found increased right frontrostriatal functional connectivity during resting state, related to higher estradiol levels during the pre-ovulatory phase^21^. The increased afferent connectivity we observed from the left hemisphere to bilateral MFG right before ovulation could reflect underlying structural changes, but in humans, the relationship between molecular mechanisms and neural functions are difficult to determine. Nevertheless, evidence from animal studies strongly suggests the involvement of oestrogen-dependent synaptogenesis in the PFC (in non-human primates^3^ and rodents^72^).

Our model shows a shift of lateralization across the cycle phases with the most evident change being the recruitment of bilateral MFG from the left insula versus the right insula during early follicular and the mid-luteal phase. In turn, the increased top-down engagement to SN from frontal ECN was maintained during the high-hormone phases, and positively related to both estradiol and progesterone levels. As expected, the right MFG increased its afferent connectivity during the mid-luteal phase, including from left MFG, which could explain the increased executive-dependent activation previously observed^37–40^. Enhanced global network connectivity have been reported before in dorsolateral PFC, associated with increased levels of progesterone^41^. Furthermore, overactivation of PFC^15^ and differential insular activity^73^ during high executive functions have been described for premenstrual dysphoric disorder (PMDD). Specifically, in patients, the left insula was found less active than in healthy controls before ovulation, while more active during the mid-luteal phase compared to both controls and pre-ovulatory patients^73^. If, as our results suggest, the balanced bottom-up/top-downregulation entails a shift in the lateralization of the dorsolateral PFC afferent connections from the SN, its absence or deregulation could underlie some symptomatology.

After ovulation, parietal areas coupled to the posterior DMN and to each other. Related to higher progesterone levels (Supplementary Data file 1), the right SMG increased the efferent connectivity to every node of the other networks but to right insula, which similarly decreased its connectivity to right SMG. A rightward asymmetry in SMG-insula connectivity has been found to be stronger in females than in males, and related to susceptibility to chronic pain disorders^74^. In addition, the right SMG is particularly involved in shifting attention to salient stimuli^75^. The shift in connectivity between the right insula and posterior ECN to anterior ECN after ovulation may reflect the dynamic integration of bottom-up/top-down processes, not only lateralized but also according to an anterior/posterior specialization. Noteworthy, all four connections with the most confident effects included in the cross-validation analysis, involved at least one node from the ECN, reflecting the key role of this network in menstrual cycle-related changes and SN/DMN coupling dynamics.

Fourth, across the menstrual cycle, two distinctive patterns regarding changes in lateralization could be distinguished. On the one hand, a right-lateralized pattern during early follicular, which changes to the left hemisphere during pre-ovulatory, and recruiting the right hemisphere again during the mid-luteal phase. This is the case for connectivity from SMG to posterior DMN, AG to SN, or the connectivity from the insula (broadly speaking). In addition, we observed an increased connectivity between homotopic regions of the SN and ECN during the mid-luteal phase. Therefore, in most cases, this time window was characterized by a decreased asymmetry, which has been previously reported for different methods including behavioural^76^, transcranial magnetic stimulation^77^ and fMRI studies^40,78^ and suggested to reflect a reduced transcallosal inhibition^79–81^. On the other hand, a more symmetrical pattern during early follicular and pre-ovulatory phase while right lateralized during the mid-luteal phase is observed for the connectivity from ECN to the SN, in which the anterior-posterior modulation makes the scenario more complex. Furthermore, and as discussed previously, the right MFG also constitutes an exception to the lateralization observed across the menstrual cycle: its connectivity to the SN was the lowest during early follicular and increased as the hormone levels increased. These differential patterns could explain why the findings involving hemispheric asymmetries are sometime controversial and depend on the specific task and cognitive system involved^82^.

In summary, this triple network dynamic model corroborates the plasticity of the brain in response to the acute ovarian hormone fluctuations along the natural menstrual cycle. We have shown how both lateralization and anterior-posterior effective connectivity patterns within and between networks depend on the endogenous hormonal status in healthy young women. Furthermore, when using those parameters with the largest posterior probability, we found an above-chance prediction of the left-out subjects’ cycle phase, although this should be interpreted with caution, given the small effect size and therefore low percentage of the variance explained. Remarkably, all these connections reflect and summarize the effects that have been already discussed: increased engagement of the DMN with the ECN during the early follicular phase, enhanced left frontoparietal connectivity right before ovulation, increased frontoparietal recruitment by the left insula in response to enhanced estradiol levels as well as interhemispheric decoupling in executive parietal areas, partially reversed after ovulation. The impact of the surge of estradiol levels around ovulation in brain connectivity and nodes flexibility^13,14^ does not come as a surprise, since the neuroendocrine feedback loops triggered by it are quite unique in the human physiology, and the biological relevance of ovulation is undeniable. Structural changes between the early follicular and the pre-ovulatory phase have been previously shown to accurately classify cycle phase using a machine learning approach, and related to estradiol levels (BrainAGE^83^). A more detailed discussion of the functional role of each of the connections included in the LOOCV can be found in the Supplementary Note 1.

The present work also corroborates a differential effect of each hormone depending on the brain region and network dynamics, which remarks the relevance of widening the focus to large-scale systems interaction, rather than the activity of localized brain areas. In fact, the cycle-related effects on connectivity from different nodes of a network to other networks suggest that ICA, the most used approach up to now, might be too coarse to capture the hormonal effects since the time-courses of various areas are averaged together and the results depend on the areas included in the ICN. Although we are still far away to comprehend the full scope of the ovarian hormones effects on the human brain coupling dynamics, this proposed causal model of menstrual cycle-related changes may improve our understanding of the underlying neuroendocrine interactions. At least to some extent, the neural substrate needs the cyclic ovarian hormones fluctuation to undergo this brain reorganization, as evidenced by animal^12,84^ and human research^13,14,85^. Therefore, results on healthy naturally cycling women may have further implications for those menstrual cycle-related disorders where this pattern of brain coupling dynamics has been suggested to be impaired, such as premenstrual syndrome (PMS), PMDD or dysmenorrhoea^19,86^. Moreover, some factors in other neurological and psychiatric disorders, where the triple model network is impaired^26^, have been previously related to hormonal levels, like the incidence of epileptic seizures^87^ addiction patterns and drug sensitivity^88^, as well as the sensitivity and risk to develop affective disorders^89,90^, Alzheimer’s disease^91^ or Schizophrenia^92,93^. Therefore, some of the key findings here described, correspondent to healthy mechanisms, may be targeted in future clinical neuroendocrine studies.

## Methods

### Participants

Seventy-eight healthy young women participated in 1 of 2 functional imaging studies^21^. Main inclusion criteria were an age range of 18–35 years, and a regular menstrual cycle (MC) of 21–35 days with a variability between cycles of <7 days^94^. Exclusion criteria included use of hormonal contraceptives within the previous 6 months, neurological, psychiatric or endocrine disorders, including PMDD and PMS and any medication intake. Due to inconsistencies between self-reported cycle phase and hormone levels, 18 women were excluded, and 2 participants were further excluded during the fMRI analysis due to insufficient signal in some of the regions of interest, resulting in a total sample of 58 healthy young women (see Table 1 for demographic information). All participants had achieved general qualification for university entrance and their IQ was measured on the Raven’s APM Screening as implemented in the Vienna Test System (WTS)^95^. All participants gave their informed written consent to participate in the study. All methods conform to the Code of Ethics by the World Medical Association (Declaration of Helsinki) and were approved by the University of Salzburg’s ethics committee.

### Procedure

Cycle duration was calculated based on participants’ self-reports of the dates of onset of their last three periods, and three appointments were scheduled once during the early follicular phase (1–7 days after the onset of current menses; low progesterone and estradiol); once in the pre-ovulatory phase (2–3 days before the expected date of ovulation; peak estradiol, low progesterone), and once during the mid-luteal phase (3 days after ovulation to 3 days before the expected onset of next menses; high progesterone and estradiol), order counter-balanced. Pre-ovulatory sessions were confirmed by commercially available urinary ovulation tests (Pregnafix®). Participants had to confirm the onset of next menses in retrospect.

### Hormone analysis

Saliva samples were collected from participants, stored and processed as described in ref. ^21^. Estradiol and progesterone were assessed using the Salimetrics High Sensitivity salivary Estradiol assay (sensitivity of 1 pg/ml) and the DeMediTec Progesterone free in saliva ELISAs (a sensitivity of 10 pg/ml), respectively. All samples were assessed in duplicates and samples with more than 25% variation between duplicates were reanalyzed. In order to corroborate hormonal changes along the menstrual cycle, statistical analyses were performed in R 3.6.2 (https://www.R-project.org/)^96^ using nlme^97^. To explore the menstrual cycle effects on estradiol and progesterone levels within-subject, a linear mixed model was fitted to the data, using estradiol and progesterone as dependent variables, cycle phase as fixed effects, and participant number (PNr) as random effect, respectively. We accounted for multiple testing by using the package multcomp^98^ for conducting all-pairwise comparisons between cycle phases. *P* values were adjusted for multiple comparisons.

### Data acquisition

Functional images, fieldmaps and an MPRAGE sequence were acquired on a Siemens Magnetom TIM Trio 3T scanner. For the resting state we used a T2*-weighted gradient echo planar (EPI) sequence with 36 transversal slices oriented parallel to the AC–PC line (whole-brain coverage, TE = 30 ms, TR = 2250 ms, flip angle 70°, slice thickness 3.0  mm, matrix 192 × 192, FOV 192 mm, in-plane resolution 2.6 × 2.6 mm). Participants were instructed to close their eyes, relax and let their mind flow. For the structural images we acquired a T1-weighted 3D MPRAGE sequence of 5 min 58 sec (160 sagital slices, slice thickness = 1 mm, TE 291 ms, TR 2300 ms, TI delay 900 ms, FA 9°, FOV 256 × 256 mm).

### Preprocessing

For the preprocessing, the first six images of each session were discarded, and functional images were despiked using 3d-despiking as implemented in AFNI (afni.nimh.nih.gov). The despiked images were then pre-processed using SPM12 standard procedures and templates SPM12 (www.fil.ion.ucl.ac.uk/spm) including segmentation of the structural images using CAT12. The resulting images were subjected to the ICA-AROMA algorithm implemented in FSL and non-aggressive removal of artefactual components^99^.

### Selection and extraction of volumes of interest

The ROIs were selected based on a large body of literature describing them as core nodes of the corresponding networks (Fig. 1a). Those included: PCC, bilateral AG and mPFC for the DMN^100,62^; bilateral AI and ACC for the SN^30,62^; and bilateral MFG and SMG for ECN^24^. The ROIs and their group-level peak coordinates are listed in Table 2 and are also shown in Fig. 1a. Following Zhou et al.^62^, the group-level peaks were identified within each ICN using spatial ICA, as implemented in the Group ICA for fMRI Toolbox (GIFT, http://mialab.mrn.org/software/gift)^101^, and already described in Hidalgo-Lopez et al.^21^. After extracting^20^ components, we selected those four corresponding to the posterior DMN (independent component; IC 19), posterior SN (IC 17), left ECN (IC 10) and right ECN (IC 11) via spatial correlation to pre-existing templates^23^ (SI, Fig. S1). Both the DMN and the SN lacked of sufficient signal in anterior regions, and therefore, the anterior nodes of the DMN (mPFC) and SN (ACC) were identified through seed-based functional connectivity analysis from the posterior nodes (as done in Razi et al.^51^) using the CONN toolbox^102^. The resulting areas were masked with BA10 & BA11 and ACC as implemented in the Wake Forest University (WFU) Pickatlas toolbox (Maldjian et al.^103^). Subject-specific coordinates were selected as local maximum within 8 mm of the group-level coordinates within the region-specific mask WFUpickatlas. For each of the 11 ROIs the principal eigenvariate from an 8 mm sphere around the subject-specific coordinates and within the ROI mask was extracted and corrected for CSF and WM (as done in Razi et al.^51^). These time series were then used in subsequent DCM analyses (Fig. 1b). Two participants were excluded due to insufficient signal in the mPFC.

**Table 2:** Group level volume of interest coordinates.

Region	MNI coordinates			Network
	X	Y	Z	
PCC	3	−55	31	DMN
lAG	−45	−64	31	DMN
rAG	46	−63	34	DMN
mPFC	0	53	−14	DMN
lAI	−39	14	−5	SN
rAI	40	11	−5	SN
ACC	0	35	19	SN
lSMG	−48	−49	37	ECN
rSMG	51	−46	37	ECN
lMFG	−42	17	31	ECN
rMFG	42	20	34	ECN

*l* left, *r* right, *PCC* precuneus/posterior cingulate cortex, *AG* angular gyrus, *mPFC* medial prefrontal cortex, *DMN* default mode network, *AI* anterior insula, *ACC* anterior cingulate cortex, *SN* salience network, *MFG* middle frontal gyrus, *SMG* supramarginal gyrus, *ECN* executive control network.

### Statistics and reproducibility

Spectral DCM for resting state was specified and inverted using DCM12 as implemented in SPM12 (www.fil.ion.ucl.ac.uk/spm). We modelled our data using a (Bayesian) hierarchical random effects model. At the first level of analysis, for each individual subject, interactions between brain regions were captured by inverting dynamic causal models (DCMs). These provided a (multivariate normal) probability density over the connectivity parameters for each subject. For each participant and session, a fully connected model (including all possible connections between nodes), with no exogenous inputs, was specified to estimate the intrinsic effective connectivity (i.e., the ‘A-matrix’) within and between networks. The estimation fits the complex cross-spectral density taking into account the effects of neurovascular fluctuations as well as noise^49^ and the default priors implemented in SPM were used at this level. The percentage of variance explained by the model for our subjects and sessions ranged from 91.60 to 99.27, which reflect good data fits for each model we estimated^104^.

For the second-level analyses, the parameters (effective connectivity strengths) were estimated in a Parametric Empirical Bayes (PEB) framework as described in Zeidman et al.^104^ The second-level model captured between-session and between-subject effects, with a general linear model (GLM) to capture effects of interest and a covariance component model to capture random effects (implemented in the Parametric Empirical Bayes framework in the SPM software, see ref. ^104^).

An advantage of this fully Bayesian approach is that models with different regressors in their group-level design matrix can be compared in terms of their log model evidence or marginal likelihood (thereby identifying the model that offers the best trade-off between accuracy and complexity). In a preliminary analysis, we evaluated whether including dummy regressors for each subject in addition to regressors for each phase of the menstrual cycle (thereby forming a within-subject repeated measures ANOVA) would increase the model evidence, relative to a model that only included regressors for each phase of the menstrual cycle. We found that the evidence decreased when including subject-specific regressors (i.e., the added complexity outweighed any increase in accuracy). Therefore, we did not include subject-specific regressors in the final regression model.

To compute the difference in effective connectivity between the three different phases, the final regression model included three regressors: first, pre-ovulatory versus early follicular; second, mid-luteal versus early follicular; and third, mid-luteal versus pre-ovulatory (Fig. 1d). The PEB analysis returned estimated effect sizes (PEB.Ep), in addition to the posterior probability (PEB.Pp) for each effect having diverged from its prior expectation of zero. In Bayesian analysis there is no need for further thresholding—there is simply the probability for each effect, with no concept of ‘significance’. Nevertheless, it can be helpful to apply thresholding in order to focus on the most probable effects. Here, we opted to use a commonly adopted definition of ‘positive evidence’^54^, by thresholding our effects at 75% posterior probability (Fig. 1). Only parameters surviving this threshold are reported in the results section and summarized in Supplementary Data file 1.

We further assessed the hormonal modulation of the connections with a second PEB analysis including scaled estradiol and progesterone levels, and their interaction as regressors (Fig. 1e). Likewise, results in Supplementary Data file 1 were thresholded to only include parameters that had a 75% posterior probability. Those connections surviving a 95% threshold for both PEB1 and PEB2 are further indicated in the figures and results section alongside each parameter’s mean and posterior probability (Fig. 1d, e). Nevertheless, given that all parameters contributed to the model, we provide figures listing all parameters regardless of threshold in the supplementary information (SI, Figs. S2 and S3).

Finally, for leave-one-out cross-validation (LOOCV), there is a strong dilution-of-evidence effect, where parameters with small effect sizes can markedly reduce predictive accuracy. It is important, therefore, to first perform feature selection, retaining only the largest or most probable parameters. Therefore, we only assessed the predictive accuracy of those parameters with 99% probability of being non-zero. In order to do so we used a leave-one-out scheme (spm_dcm_loo.m) as described in Friston et al.^50^ and tested whether the effect on these particular connections could predict the cycle phase of participants.

### Reporting summary

Further information on research design is available in the Nature Research Reporting Summary linked to this article.

## Supplementary information

Supplementary Information

Description of Additional Supplementary Files

Supplementary Data 1

Supplementary Data 2

Supplementary Data 3

Reporting Summary

## Data Availability

Data and scripts are openly available online at http://webapps.ccns.sbg.ac.at/OpenData/ and OSF, https://osf.io/23d7x/^105^. MR-images are available upon request from the first author. A summary of the results is provided in Supplementary Data file 1 and source data for Fig. 4b and c are provided in Supplementary Data file 2 and 3, respectively.
