# Supplementary material for: Spectral dynamic causal modelling in healthy women reveals brain connectivity changes along the menstrual cycle
Source: Commun Biol. 2021 Aug 10;4:954. doi: 10.1038/s42003-021-02447-w (PMC8355156; doi:10.1038/s42003-021-02447-w)
Supplement: Supplementary file 1 — Supplementary Information [file 42003_2021_2447_MOESM1_ESM.pdf]

Spectral dynamic causal modelling in healthy women reveals brain connectivity changes along the menstrual cycle

Esmeralda Hidalgo-Lopez[1*], Peter Zeidman[2], TiAnni Harris[1], Adeel Razi[2,3], Belinda Pletzer[1*]

[1]Department of Psychology and Centre for Cognitive Neuroscience,

University of Salzburg, Hellbrunnerstr. 34, 5020 Salzburg, Austria.

[2]The Wellcome Centre for Human Neuroimaging, University College London,

Queen Square, London WC1N 3AR, United Kingdom

[3]Turner Institute for Brain and Mental Health, Monash University, Clayton, Victoria, Australia

***Corresponding authors:**

Esmeralda Hidalgo-Lopez: esmeralda.hidalgolopez@sbg.ac.at (Hidalgo-Lopez, E.)

Hellbrunnerstr. 34, 5020 Salzburg

0043-662-8044-5178

Belinda Pletzer: belinda.pletzer@gmail.com (Pletzer, B.)

Hellbrunnerstr. 34, 5020 Salzburg

0043-662-8044-5184

SUPPLEMENTARY INFORMATION

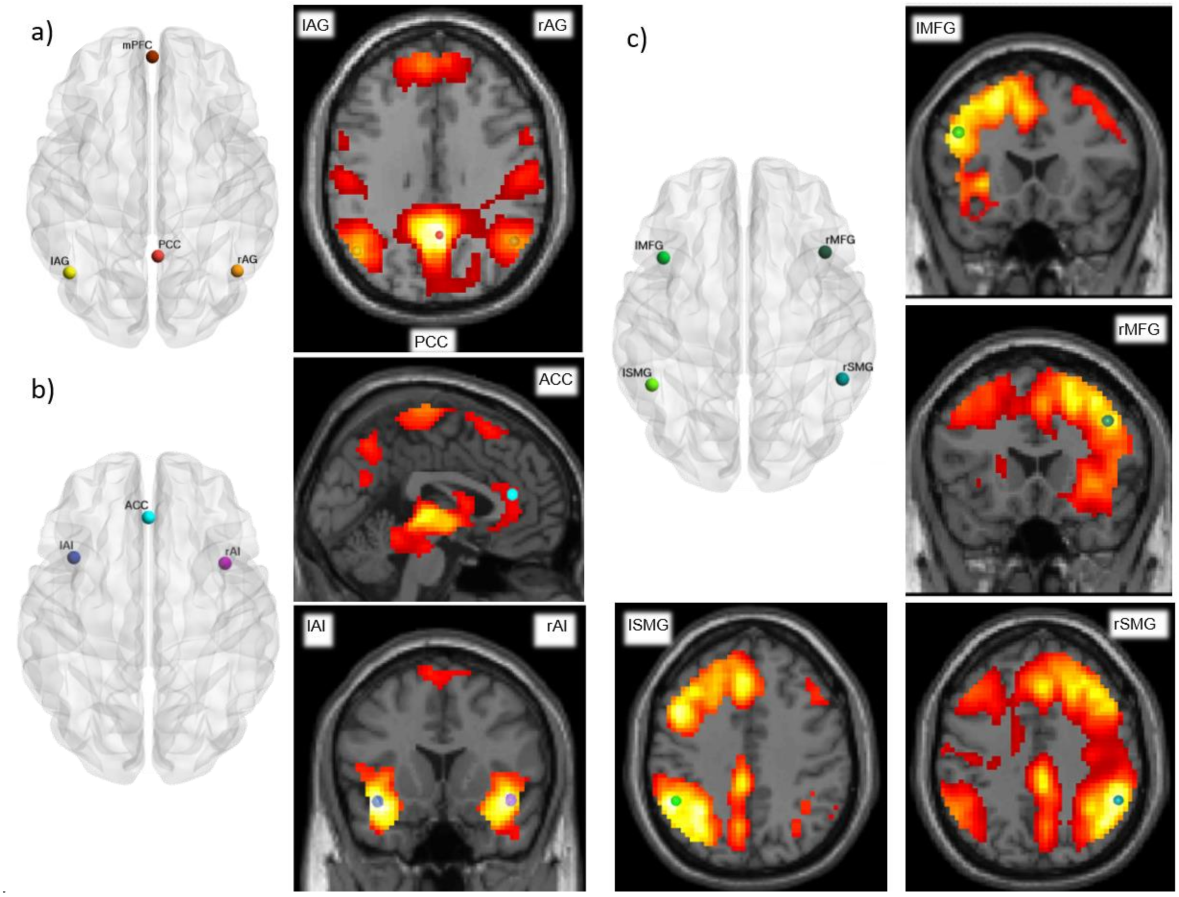

Fig. S1: Group-level ROIs identified using spatial independent component analysis (ICA). The group-level peak coordinates are overlaid on the spatial distribution maps from the ICNs of interest, correspondent to the a) DMN, b) SN, c) left ECN and right ECN, as spatially matched to pre-existing templates from Laird et al., 2011.

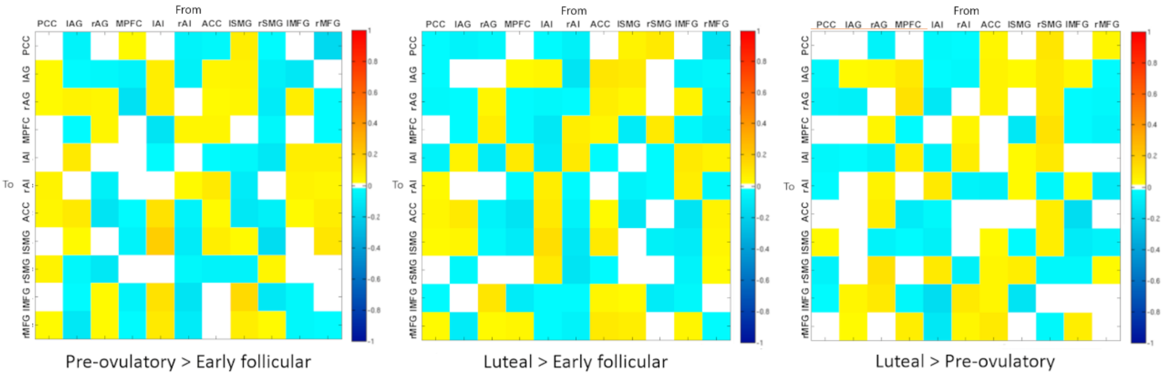

Fig. S2: Effective connectivity matrix reflecting differences in effective connectivity within and between intrinsic connectivity networks DMN, SN and ECN across the menstrual cycle. The columns are the outgoing connections, the rows are the incoming connections, ordered as: PCC, lAG, rAG, mPFC, lAI, rAI, ACC, lSMG, rSMG, lMFG, and rMFG. Hot colours indicate positive parameter estimates and cold colours negative Every parameter regardless of threshold is displayed, given that all parameters contributed to the model. A) Pre-ovulatory vs. early follicular, b) luteal vs. early follicular, c) luteal vs. pre-ovulatory.

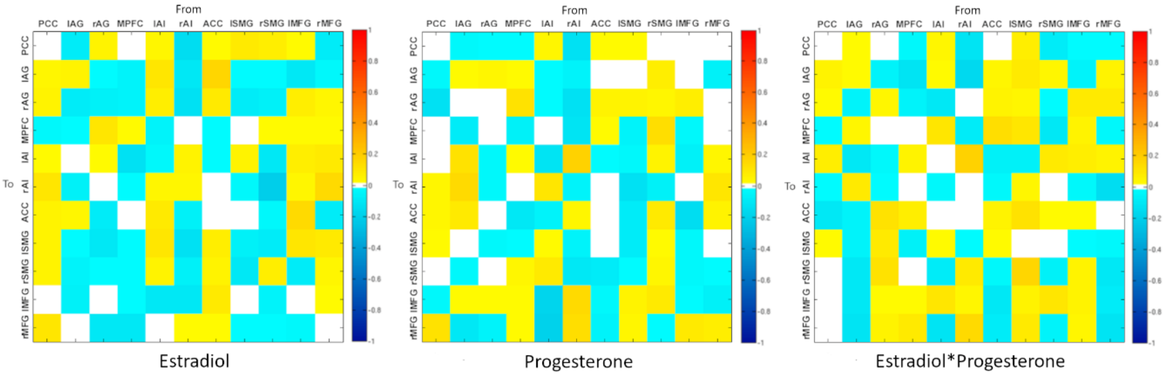

Fig. S3: Effective connectivity matrix reflecting differences in effective connectivity within and between intrinsic connectivity networks DMN, SN and ECN related to hormone levels a) estradiol, b) progesterone, and c) its interaction. The columns are the outgoing connections, the rows are the incoming connections, ordered as: PCC, lAG, rAG, mPFC, lAI, rAI, ACC, lSMG, rSMG, lMFG, and rMFG. Hot colours indicate positive parameter estimates and cold colours negative. Every parameter regardless of threshold is displayed, given that all parameters contributed to the model.

Supplementary Note 1

From left insula to the left SMG: The role of the insula identifying relevant internal and external stimuli and recruiting frontoparietal areas to guide behaviour would be essential right before ovulation for reproductive purposes. Relatedly, estradiol modulates temporal decision-making and impulsivity [1] which has been associated to the insula activity. As previously described, healthy women showed increased left insula activation during response inhibition right before ovulation compared to premenstrual dysphoric disorder patients [2]. The increase of effective connectivity from the left insula to the left SMG, could explain changes in impulsivity that have been observed before ovulation for some women. In a previous work, we found interindividual differences in left putamen connectivity to ACC and left SMG right before ovulation, and related to changes in inhibitory control across the menstrual cycle [3]. Given that striatal-insula functional connectivity is related to impulsivity trait [4], the present model would place the left insula as intermediate between subcortical and cortical structures. The enhanced efferent connections of the left insula during the pre-ovulatory phase could explain the absence of interindividual differences in BOLD-activation, but opposite patterns of connectivity between left striatum, ACC and left SMG depending on the impulsiveness of women.

From left SMG to left MFG: In turn, the left SMG increased its connectivity to the left MFG. Both areas are structurally connected by the superior longitudinal fasciculus, previously reported to vary depending on sex hormones levels [5]. Interestingly, both areas are the structural underpinnings of the human mirror system, and related to language and verbal abilities [6,7]. The mirror system plays an important role in social cognition and emotional empathy, and its sex differences have been suggested to depend on the hormonal milieu [7]. Higher accuracy in emotion discrimination and recognition has been related to increased connectivity and activation within the mirror system [7]. The increased input in left MFG from left parietal areas during the pre-ovulatory phase, caused in turn by the left insula, could underlie a general better emotion recognition described for this phase [8]. Likewise, given that the left MFG corresponds to the Broca's area, the increased connectivity could also underlie improved verbal abilities related to higher hormonal levels (for a review, see Luine, 2014; Sherwin, 2012).

From right MFG to PCC: On the other hand, the right MFG disengaged from the PCC in response to the rise of estradiol levels, which allows the later to couple to posterior areas of DMN and ECN. Dorsolateral PFC and PCC are strongly interconnected and have been suggested to underlie (specially the right hemisphere) the necessary associative processes for self-awareness, self-representation and conscious experience [10]. The effective connectivity between these areas has already been reported to be modulate by endocrine factors in males, decreasing from dorsolateral PFC to PCC after oxytocin treatment [11]. In women, structural changes in the PCC related to the hormonal milieu have also been reported, with decreased cortical thickness in oral contraceptive users [12].

From right SMG to left SMG: Finally, while the driving input for left SMG during the early follicular phase is its homotopic region, the rise in estradiol levels causes an interhemispheric decoupling, allowing the left hemisphere to increase its within connectivity. The SMG is involved in cross-modal integration processes, comprising a wide range of functions such as proprioception, calculation, social cognition, or praxis functions. In the later, a left predominance for planning and verbal communication, while right one for visuospatial processing is well-known. For the execution of the action itself, the lateralization is more diffused [13]. Menstrual cycle-related changes in the

connectivity between homotopic SMG could underlie the differential effect of sex and hormonal status during visuo-spatial tasks. During the early follicular phase, women showed more activation in the left SMG, and did not differ behaviourally from men [14]. After ovulation, progesterone counteracted estradiol, and the homotopic connectivity increased again, contributing to a decreased asymmetry.

Supplementary references

1. Diekhof, E. K. Be quick about it. Endogenous estradiol level, menstrual cycle phase and trait impulsiveness predict impulsive choice in the context of reward acquisition. *Horm. Behav.* **74**, 186–193 (2015).

2. Bannbers, E. *et al.* The effect of premenstrual dysphoric disorder and menstrual cycle phase on brain activity during response inhibition. *J. Affect. Disord.* **142**, 347–350 (2012).

3. Hidalgo-Lopez, E. & Pletzer, B. Individual differences in the effect of menstrual cycle on basal ganglia inhibitory control. *Sci. Rep.* **9**, 11063 (2019).

4. McHugh, M. J. *et al.* Striatal-insula circuits in cocaine addiction: Implications for impulsivity and relapse risk. *Am. J. Drug Alcohol Abuse* (2013) doi:10.3109/00952990.2013.847446.

5. Herting, M. M., Maxwell, E. C., Irvine, C. & Nagel, B. J. The impact of sex, puberty, and hormones on white matter microstructure in adolescents. *Cereb. Cortex* (2012) doi:10.1093/cercor/bhr246.

6. Caspers, S., Zilles, K., Laird, A. R. & Eickhoff, S. B. ALE meta-analysis of action observation and imitation in the human brain. *Neuroimage* (2010) doi:10.1016/j.neuroimage.2009.12.112.

7. Farina, E., Borgnis, F. & Pozzo, T. Mirror neurons and their relationship with neurodegenerative disorders. *Journal of Neuroscience Research* (2020) doi:10.1002/jnr.24579.

8. Poromaa, I. S. & Gingnell, M. Menstrual cycle influence on cognitive function and emotion processing from a reproductive perspective. *Front. Neurosci.* **8**, 380 (2014).

9. Luine, V. N. Estradiol and cognitive function: Past, present and future. *Horm. Behav.* **66**, 602–618 (2014).

10. Cavanna, A. E. & Trimble, M. R. The precuneus: A review of its functional anatomy and behavioural correlates. *Brain* (2006) doi:10.1093/brain/awl004.

11. Kumar, J., J. Iwabuchi, S., A. Völlm, B. & Palaniyappan, L. Oxytocin modulates the effective connectivity between the precuneus and the dorsolateral prefrontal cortex. *Eur. Arch. Psychiatry Clin. Neurosci.* (2019) doi:10.1007/s00406-019-00989-z.

12. Petersen, N., Touroutoglou, A., Andreano, J. M. & Cahill, L. Oral contraceptive pill use is associated with localized decreases in cortical thickness. *Hum. Brain Mapp.* (2015) doi:10.1002/hbm.22797.

13. Bohlhalter, S. *et al.* Gesture subtype-dependent left lateralization of praxis planning: An event-related fMRI study. *Cereb. Cortex* (2009) doi:10.1093/cercor/bhn168.

14. Schöning, S. *et al.* Functional anatomy of visuo-spatial working memory during mental rotation is influenced by sex, menstrual cycle, and sex steroid hormones. *Neuropsychologia* **45**, 3203–3214 (2007).
