# Supplementary material for: Spectral dynamic causal modelling in healthy women reveals brain connectivity changes along the menstrual cycle
Source: Commun Biol. 2021 Aug 10;4:954. doi: 10.1038/s42003-021-02447-w (PMC8355156; doi:10.1038/s42003-021-02447-w)
Supplement: Supplementary file 2 — Description of Additional Supplementary Files [file 42003_2021_2447_MOESM2_ESM.pdf]

Description of Additional Supplementary Files

File Name: Supplementary Data 1
Description: **Summary of connections that showed cycle phases differences organized by hormonal relations.** Columns 2-4 correspond to cycle phase differences that survived a 75% posterior probability threshold. The hormonal modulation of these connections are detailed in columns 5-7, indicated by a + if relationship to the connectivity parameter was positive, and - if the relationship was negative. Connections that were able to predict cycle phase are marked in bold. EF: early follicular, P-O: pre-ovulatory, L: luteal, E: estradiol, P: progesterone, E*P: estradiol and progesterone interaction

File Name: Supplementary Data 2
Description: **Source data for Fig.4b**. For displaying purposes, the individual values of each parameter with a posterior probability > 0 .99 were extracted for each cycle phase.

File Name: Supplementary Data 3
Description: **Source data for Fig.4c.** Out-of-samples correlation from the LOOCV analysis displaying the correlation between the actual cycle phase in the leftout-subject's design matrix (early follicular, pre-ovulatory or mid-luteal) and the predicted cycle phase based on the left-outsubject's connectivity.
