# Supplementary material for: Spectral dynamic causal modelling in healthy women reveals brain connectivity changes along the menstrual cycle
Source: Commun Biol. 2021 Aug 10;4:954. doi: 10.1038/s42003-021-02447-w (PMC8355156; doi:10.1038/s42003-021-02447-w)
Supplement: Supplementary file 6 — Reporting Summary [file 42003_2021_2447_MOESM6_ESM.pdf]

nature research

Corresponding author(s): Esmeralda Hidalgo-Lopez & Belinda Pletzer

Last updated by author(s): Jun 25, 2021

Reporting Summary

Nature Research wishes to improve the reproducibility of the work that we publish. This form provides structure for consistency and transparency in reporting. For further information on Nature Research policies, see our Editorial Policies and the Editorial Policy Checklist.

Statistics

For all statistical analyses, confirm that the following items are present in the figure legend, table legend, main text, or Methods section.

n/a	Confirmed	
☐	☒	The exact sample size (*n*) for each experimental group/condition, given as a discrete number and unit of measurement
☐	☒	A statement on whether measurements were taken from distinct samples or whether the same sample was measured repeatedly
☐	☒	The statistical test(s) used AND whether they are one- or two-sided<br>*Only common tests should be described solely by name; describe more complex techniques in the Methods section.*
☐	☒	A description of all covariates tested
☐	☒	A description of any assumptions or corrections, such as tests of normality and adjustment for multiple comparisons
☐	☒	A full description of the statistical parameters including central tendency (e.g. means) or other basic estimates (e.g. regression coefficient) AND variation (e.g. standard deviation) or associated estimates of uncertainty (e.g. confidence intervals)
☐	☒	For null hypothesis testing, the test statistic (e.g. F, t, r) with confidence intervals, effect sizes, degrees of freedom and P value noted<br>*Give P values as exact values whenever suitable.*
☐	☒	For Bayesian analysis, information on the choice of priors and Markov chain Monte Carlo settings
☐	☒	For hierarchical and complex designs, identification of the appropriate level for tests and full reporting of outcomes
☐	☒	Estimates of effect sizes (e.g. Cohen's d, Pearson's r), indicating how they were calculated

Our web collection on statistics for biologists contains articles on many of the points above.

Software and code

Policy information about availability of computer code

Data collection	Neuroimaging data were acquired on a Siemens Magnetom Trio Tim 3 Tesla scanner.
Data analysis	MRI-data was analyzed using Statistical Parametric Mapping package (SPM12, https://www.fil.ion.ucl.ac.uk/spm/software/spm12). To corroborate hormonal changes along the menstrual cycle, statistical analyses were performed in R 3.6.2 (https://www.R-project.org/).

For manuscripts utilizing custom algorithms or software that are central to the research but not yet described in published literature, software must be made available to editors and reviewers. We strongly encourage code deposition in a community repository (e.g. GitHub). See the Nature Research guidelines for submitting code & software for further information.

Data

Policy information about availability of data

All manuscripts must include a data availability statement. This statement should provide the following information, where applicable:

- Accession codes, unique identifiers, or web links for publicly available datasets
- A list of figures that have associated raw data
- A description of any restrictions on data availability

Data and scripts are openly available online at http://webapps.ccns.sbg.ac.at/OpenData/ and OSF, https://osf.io/23d7x/. MR-images are available upon request from the first author. A summary of the results is provided in Supplementary Data file 1 and source data for figures 4.b and c are provided in Supplementary Data file 2 and 3, respectively.

Field-specific reporting

Please select the one below that is the best fit for your research. If you are not sure, read the appropriate sections before making your selection.

- [x] Life sciences
- [ ] Behavioural & social sciences
- [ ] Ecological, evolutionary & environmental sciences

For a reference copy of the document with all sections, see nature.com/documents/nr-reporting-summary-flat.pdf

Life sciences study design

All studies must disclose on these points even when the disclosure is negative.

Sample size	Final sample of 58 healthy right-handed women aged 18-35, thrice scanned each. Power simulations for the functional analysis were run using the longpower package in R.
Data exclusions	Main inclusion criteria were an age range of 18–35 years, and a regular menstrual cycle (MC) of 21–35 days with a variability between cycles of less than 7 days. Exclusion criteria included use of hormonal contraceptives within the previous 6 months, neurological, psychiatric or endocrine disorders, including premenstrual dysphoric disorder (PMDD) and premenstrual syndrome (PMS) and any medication intake. Due to inconsistencies between self-reported cycle phase and hormone levels, 18 women were excluded, and 2 participants were further excluded during the fMRI analysis due to insufficient signal in some of the regions of interest, resulting in a total sample of 58 healthy young women.
Replication	N/A
Randomization	Participans were tested three times and the mentrual cycle phase in which they started was counterbalanced.
Blinding	Investigators were not blinded to group allocation during data collection and analysis.

Reporting for specific materials, systems and methods

We require information from authors about some types of materials, experimental systems and methods used in many studies. Here, indicate whether each material, system or method listed is relevant to your study. If you are not sure if a list item applies to your research, read the appropriate section before selecting a response.

Materials & experimental systems

n/a	Involved in the study	
x		Antibodies
x		Eukaryotic cell lines
x		Palaeontology and archaeology
x		Animals and other organisms
	x	Human research participants
x		Clinical data
x		Dual use research of concern

Methods

n/a	Involved in the study	
x		ChIP-seq
x		Flow cytometry
	x	MRI-based neuroimaging

Human research participants

Policy information about studies involving human research participants

Population characteristics	Fifty-eight women, with a mean age of 25.36 ±0.56 years old, and IQ=110.31±1.21. Demographics are detailed in Table 1.
Recruitment	Participants were recruited via advertisement (broadcast emailing & posters) at the Faculty of Natural Science (University of Salzburg).
Ethics oversight	University of Salzburg's ethics committee

Note that full information on the approval of the study protocol must also be provided in the manuscript.

Magnetic resonance imaging

Experimental design

Design type	Resting state.
Design specifications	Resting state scan of about 9 min duration.

Behavioral performance measures	N/A

Acquisition

Imaging type(s)	Functional & structural
Field strength	3T
Sequence & imaging parameters	Functional: T2*-weighted gradient echo planar (EPI) sequence with 36 transversal slices oriented parallel to the AC–PC line (whole-brain coverage, TE=30 ms, TR=2250 ms, flip angle 70°, slice thickness 3.0 mm, matrix 192×192, FOV 192 mm, in-plane resolution 2.6×2.6 mm).<br>Structural: T1-weighted 3D MPRAGE sequence (160 sagital slices, slice thickness=1 mm, TE 291 ms, TR 2300 ms, TI delay 900 ms, FA 9°, FOV 256×256 mm).
Area of acquisition	Whole-brain.

Diffusion MRI ☐ Used ☒ Not used

Preprocessing

Preprocessing software	AFNI for 3d-despiking (afni.nimh.nih.gov). SPM12 for realignment, co-registration, normalization and smoothing, CAT12 for segmentation, and ICA-AROMA for non-aggressive removal of artefactual components.
Normalization	Structural images were segmented and normalized using the computational anatomy toolbox (CAT12).
Normalization template	MNI152
Noise and artifact removal	3d-despiking, realignment, ICA-AROMA algorithm implemented in FSL and non-aggressive removal of artefactual components.
Volume censoring	N/A

Statistical modeling & inference

Model type and settings	We modelled our data using a (Bayesian) hierarchical random effects model. At the first level of analysis, for each individual subject, interactions between brain regions were captured by inverting dynamic causal models (DCMs). These provided a (multivariate normal) probability density over the connectivity parameters for each subject. For each participant and session, a fully connected model (including all possible connections between nodes), with no exogenous inputs, was specified to estimate the intrinsic effective connectivity (i.e., the 'A-matrix') within and between networks. Default priors implemented in SPM were used at this level. For the second level analyses, the parameters (effective connectivity strengths) were estimated in a Parametric Empirical Bayes (PEB) framework as described in Zeidman et al. (2019). For the group level priors the PEB takes the average of the DCM priors over subjects.
Effect(s) tested	The second level model captured between-session and between-subject effects, with a general linear model (GLM) to capture effects of interest and a covariance component model to capture random effects. We found that the evidence decreased when including subject-specific regressors (i.e., the added complexity outweighed any increase in accuracy). Therefore, we did not include subject-specific regressors in the final regression model.<br>To compute the difference in effective connectivity between the three different phases, the final regression model included three regressors: first, pre-ovulatory versus early follicular; second, mid-luteal versus early follicular; and third, mid-luteal versus pre-ovulatory. We further assessed the hormonal modulation of the connections with a second PEB analysis including scaled estradiol and progesterone levels, and their interaction as regressors.

Specify type of analysis: ☐ Whole brain ☒ ROI-based ☐ Both

Anatomical location(s)	The ROIs were selected based on a large body of literature describing them as core nodes of the default mode network (DMN), salience network (SN), and executive control network (ECN). Thus, ROIs included: precuneus/posterior cingulate cortex (PCC), bilateral angular gyri (AG) and medial prefrontal cortex (mPFC) for the DMN (Di and Biswal, 2014; Zhou et al., 2018); bilateral anterior insula (AI) and anterior cingulate cortex (ACC) for the SN (Seeley et al., 2007; Zhou et al., 2018); and bilateral middle frontal gyri (MFG) and supramarginal gyri (SMG) for ECN (Damoiseaux et al., 2006). Group-level peaks were identified within each intrinsic connectivity network (ICN) using spatial ICA, and through seed-based functional connectivity analysis using the CONN toolbox (as done in Razi et al., 2015). For each of the 11 ROIs the principal eigenvariate from an 8 mm sphere around the subject-specific coordinates and within the ROI mask as implemented in the Wake Forest University (WFU) Pickatlas toolbox (Maldjian et al., 2003).

Statistic type for inference
(See Eklund et al. 2016)

The PEB analysis returned estimated effect sizes (PEB.Ep), in addition to the posterior probability (PEB.Pp) for each effect having diverged from its prior expectation of zero. In Bayesian analysis there is no need for further thresholding – there is simply the probability for each effect, with no concept of 'significance'. Nevertheless, it can be helpful to apply thresholding in order to focus on the most probable effects. Here, we opted to use a commonly adopted definition of 'positive evidence' (Kass and Raftery, 1995), by thresholding our effects at 75% posterior probability. As suggested by one of the reviewers, those connections surviving a 95% threshold for both PEB1 and PEB2 are further indicated in the figures and results section. A threshold of p<0.05 was usd for the cross-validation analysis of those connections that survived a 99% posterior probability.
Kass, R.E., Raftery, A.E., 1995. Bayes Factors. J. Am. Stat. Assoc. https://doi.org/10.2307/2291091

Correction

Instead of Monte Carlo method, DCM uses Variational Bayes under the Laplace Approximation. The settings were the default implemented in the SPM / DCM software.

Models & analysis

n/a	Involved in the study
☐	☒ Functional and/or effective connectivity
☒	☐ Graph analysis
☐	☒ Multivariate modeling or predictive analysis

Functional and/or effective connectivity

At the first level of analysis, spectral dynamic causal models (DCMs) provided a (multivariate normal) probability density over the connectivity parameters for each subject. For this first level the percentage of variance explained by the model for our subjects and sessions was above 90%. At the second level, PEB analysis returned estimated effect sizes (PEB.Ep), in addition to the posterior probability (PEB.Pp) for each effect having diverged from its prior expectation of zero. PEB results were thresholded to only include parameters from the A matrix that had more than 75% posterior probability, and Ep and Pp are included in the results, indicating those greater then 95%.

Multivariate modeling and predictive analysis

For the leave-one-out cross-validation (LOOCV), we first perform feature selection, retaining only the largest or most probable parameters. Therefore, we only assessed the predictive accuracy of those parameters with 99% probability of being non-zero. Pearson's correlation coefficient was calculated between the actual cycle phase in the left-out-subject's design matrix (early follicular, pre-ovulatory or mid-luteal) and the predicted cycle phase based on the left-out-subject's connectivity. For this analysis we used a leave-one-out scheme (spm_dcm_loo.m) as described in Friston et al. (2016).
